# Evaluation of Bait Attractiveness for *Vespa orientalis* and *Vespa crabro* (Hymenoptera: Vespidae) in Urban and Apiary Environment of Campania Region (Italy)

**DOI:** 10.3390/insects17040368

**Published:** 2026-03-31

**Authors:** Manuela Martano, Karen Power, Serena Montagnaro, Marco Esposito, Claudia D’Emilio, Paola Maiolino

**Affiliations:** 1 Department of Veterinary Medicine and Animal Production, University of Naples Federico II, Via Delpino, 80137 Naples, Italy; manuela.martano@unina.it (M.M.); serena.montagnaro@unina.it (S.M.); claudia.demilio@unina.it (C.D.); maiolino@unina.it (P.M.); 2 Department of Biology, University of Naples Federico II, Via Cinthia, 80126 Naples, Italy; 3 UOS Prevenzione e Sanità Pubblica Veterinaria—Regione Campania, Centro Direzionale is. C3, 80143 Naples, Italy; marco.esposito@regione.campania.it; 4 NBFC, National Biodiversity Future Center, 90133 Palermo, Italy

**Keywords:** apiaries, baited traps, *Vespa crabro*, *V. orientalis*, urban environments

## Abstract

*Vespa orientalis* is an emerging pest in southern Italy, with increasing impacts on bees in apiculture and urban environments. This study assessed the attractiveness of three bait types—beer, canned peaches, and commercial fish-based cat food—used in transparent plastic bottle traps deployed in apiary and urban sites across the Campania region. Trapping was conducted from September to October in 15 apiaries and 10 urban areas. A total of 419 hornets (*V. orientalis* and *V. crabro*) were collected. *V. crabro* showed a strong preference for beer-baited traps, whereas *V. orientalis* did not exhibit significant bait preferences, although most captures occurred in protein-baited traps. Species composition differed between environments, with *V. orientalis* more prevalent in urban sites. Overall, low capture rates of *V. orientalis* despite high presence in apiaries suggest that conventional attractant-based trapping is poorly suited for this species, highlighting the need for behaviour-based monitoring tools.

## Simple Summary

The oriental hornet *Vespa orientalis* is increasingly reported in southern Italy, where it threatens honey bee colonies and is becoming more common in urban areas. This study evaluated the attractiveness of three easily available food baits—beer, canned peaches, and fish-based cat food—used in plastic bottle traps placed in apiaries and urban sites across the Campania region during September–October 2025. A total of 419 hornets were captured, including *V. orientalis* and *V. crabro*. While *V. crabro* showed a clear preference for beer-baited traps, *V. orientalis* did not exhibit a significant attraction to any specific bait, although slightly more individuals were captured in protein-based traps. The two species also showed different spatial distributions: *V. crabro* was more common in apiaries, whereas *V. orientalis* predominated in urban sites. Despite frequent reports of predation on honey bees, the relatively low capture rate of *V. orientalis* suggests that traditional baited traps may not be sufficiently effective for monitoring this species. More behaviour-based monitoring strategies are therefore needed to improve management in both apicultural and urban contexts.

## 1. Introduction

Since ancient times, bees are the most widespread managed pollinator [1]. Across the world, beekeeping represents a valuable activity under the environmental, social and economic point of view as it sustains pollination, creates job opportunities and provides income to many families [2,3]. The Italian beekeeping market is composed mainly by professional beekeepers for which beekeeping represents the main source of income with approximately 50 colonies per activity [4]. It appears clear that for Italian beekeepers the loss of colonies due to different stressors could represent a serious threat to the economic stability of the family. Among the factors which can challenge the health of honey bee colonies, extreme weather conditions, pesticides and pathogens represent the most frequently associated with colony loss [5]; however, more recently, honey bee predators of the Vespidae family are raising many concerns in the beekeeping field [6,7].

Currently five different species of *Vespa* sp. have been reported in Europe: *V. orientalis*, *V. velutina*, *V. soror*, *V. bicolor* and *V. crabro*, the only species considered native to most European countries [8,9], including Italy, where it does not represent a great threat for apiculture probably due to its lower preference for honey bees [10]. On the contrary, *V. velutina* and *V. orientalis* represent, respectively, an everyday challenge for northern and southern Italian beekeepers.

*Vespa orientalis* (Linnaeus, 1771) is a hornet species (Hymenoptera: Vespidae) native to the southeastern Mediterranean region, northeastern and eastern Africa, the Middle East, and the southern part of southwestern Asia [11,12,13,14,15]. Given its origin, it is commonly known as the “Oriental hornet.” Over recent years, *V. orientalis* has expanded its distribution range in several new non-native territories [16,17,18,19], due to involuntary anthropic introduction, climate change, and habitat loss, which can reduce available nesting sites and food supplies for hornets [20,21]. It has significantly extended its distribution to other European countries [22,23,24,25,26,27], with records from eastern and southern Spain [6,22,23,24,25,26,27], Romania [27], southern France [26], and the Greek islands [28]. Climate-niche analyses indicate that *V. orientalis* is well adapted to warm temperatures and low-precipitation regimes, which facilitate its spread across semi-arid and Mediterranean climates [29]. In Italy, *V. orientalis* is well established in Sicily, Calabria and Campania. However, the species has recently expanded its range into central and northern regions of the country [30,31]. In 2021, a single adult specimen was reported from Sardinia (Cagliari) (www.stopvelutina.it), while a considerable presence of *V. orientalis* has been documented in Latium, Liguria, Tuscany [31], Marche and Trieste [30].

*V. orientalis* is recognizable by its rusty-red body coloration, yellow bands on the metasomal segments, and a distinctive yellow mark on the face [32]. Colonies are annual and originate from a single queen, which founds the nest in spring after winter diapause [33,34]. Colony size increases during spring and summer, reaching peaks of 4000 individuals in late summer/beginning of autumn, before declining with decreasing temperatures [35]. Reproduction occurs between September and December when new queens and drones emerge and mate, after which fertilised queens search for appropriate hibernation space [36]. However, in recent years a shift in the life cycle has been observed with colony peak and reproduction occurring later in time. Feeding behaviour varies with age: workers mainly consume carbohydrates such as fruit nectar and honey, whereas larvae require protein sources, often obtained from honey bees [11,22,35,37]. Larvae, for their part, process the proteins ingested and return them to the queen as a drop of saliva composed of carbohydrates and free amino acids [38].

Expansion of *Vespa* sp. populations in *Apis mellifera* apiaries may lead to increased honey bee mortality and reduced hive productivity, posing a serious threat to apiculture. *V. orientalis* attacks honey bee foragers upon returning to the hive after the foraging flights, reducing their foraging activity [36]. By plundering honey, pollen, and larvae from hives *V. orientalis* further weakens honey bee colonies [39,40]. Reduction in foraging activity, combined with a scarcity of food storage, could result in inadequate nourishment of honey bees [41,42], making them more susceptible to the effects of pesticides and pathogens [43]. Moreover, the predation of infected honey bees by hornets may facilitate the transmission of bee viruses to *V. orientalis,* representing a potential vector of honey bee pathogens [44,45]. Recent reports indicate a significant expansion of *V. orientalis* in urban areas, likely due to the urban “heat island effect” and the presence of green urban spaces such as domestic gardens, botanical gardens, food gardens, and urban apiaries, which provide abundant food resources and shelter for many insects [46,47]. The expansion of *V. orientalis* not only harms honey bee populations, but can pose risks to human safety, although *V. orientalis* appears not to be particularly aggressive toward humans when away from the nest [48,49]. In the Campania region, the rise in *V. orientalis* populations has been associated with an increase in stings and allergic reactions from encounters with both *V. orientalis* and other hornet species (In 2023, the project “Emergency *Vespa Orientalis* in Campania (EVOC)” was founded to evaluate the presence of *V. orientalis* in the Campania region and propose possible mitigation strategies). Bait trapping is one of the most used tools to capture wasps and hornets, working both as a monitoring and a controlling tool [50,51,52]. While bait trapping is often used to detect the presence of *Vespa velutina* [53,54], few studies have been conducted on trapping techniques for *V. orientalis* [37,55,56,57].

This study aimed to evaluate the attractiveness of *V. orientalis* to three different trap baits localised in both apiary and urban environments across the Campania region. Therefore, two carbohydrate-based baits (beer and canned peaches) and one protein-based bait (commercial cat food) were deployed in plastic bottle traps to evaluate their effectiveness as monitoring and capturing tools for *V. orientalis*. The results are expected to inform targeted management strategies and contribute to the development of more effective control measures for *V. orientalis*.

## 2. Materials and Methods

### 2.1. Sampling Sites

**Figure 1:**
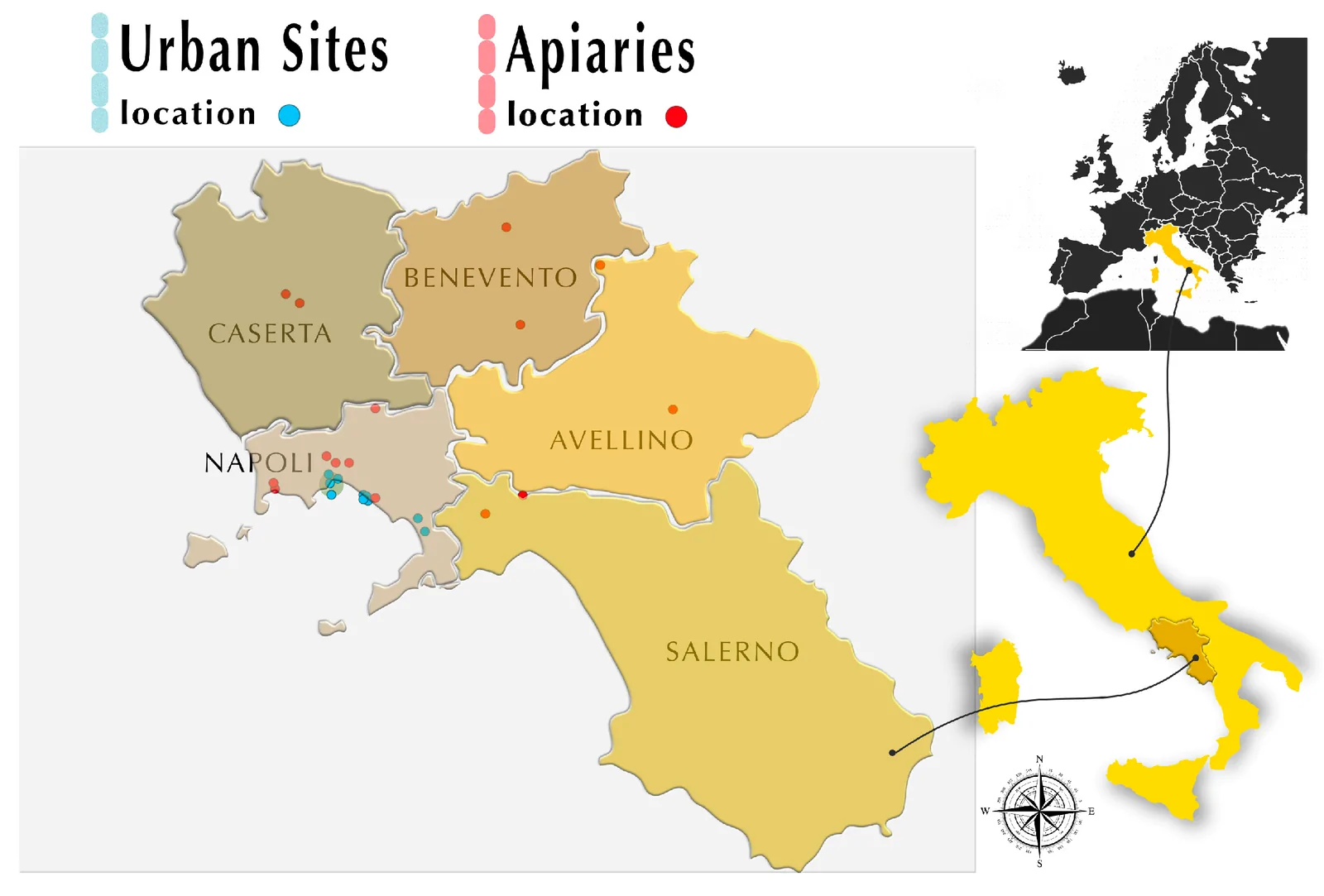
Map of the Campania region showing the location of monitored urban sites (blue) and apiaries (red) included in the study.

From early September to late October 2025, according to the aims of the EVOC project, bottle traps were installed in fifteen apiaries distributed across the Campania region (Figure 1, Table S1), as well as in ten urban sites within the city of Naples (Table S2), the latter selected based on the presence of large, heavily frequented public green areas. Each site was labelled, coded, and geo-referenced and information on the surrounding landscape was collected to classify sites as rural apiaries or urban/suburban locations. The trap-setting period was determined according to previous studies reporting highest hornet density and activity between late summer and early autumn [15].

### 2.2. Traps and Baits

**Figure 2:**
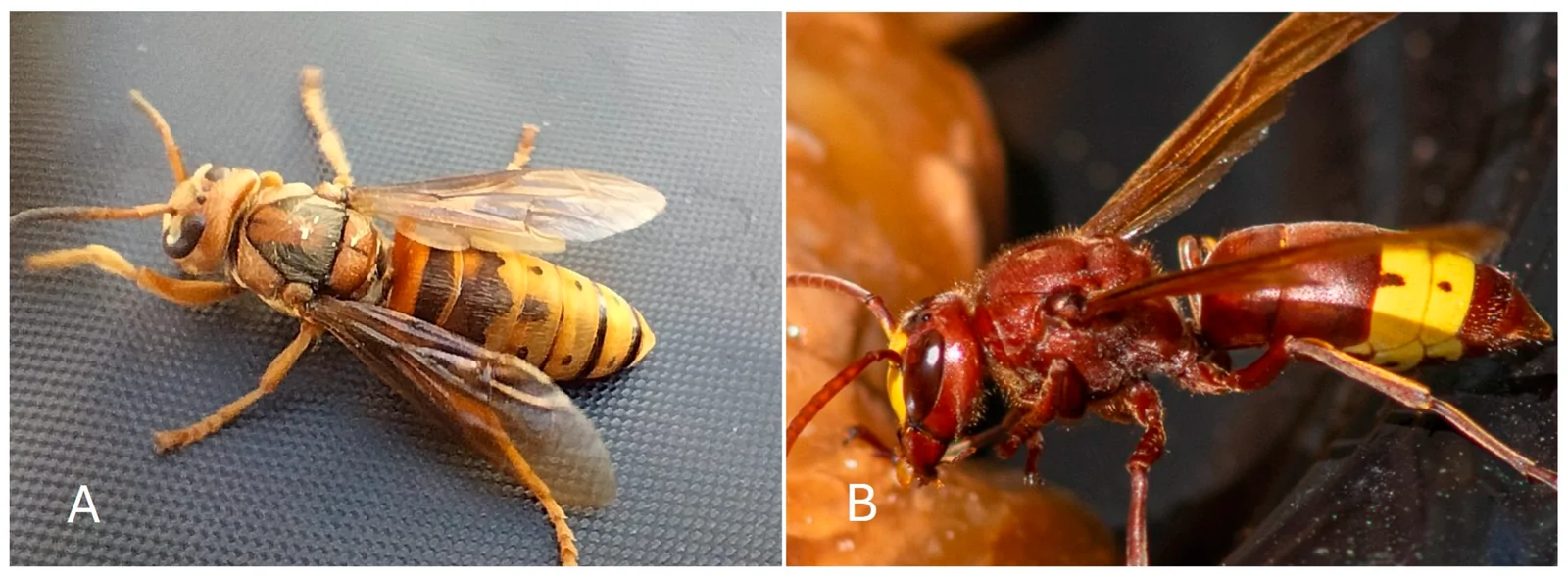
Morphological comparison of hornets: (**A**) *Vespa crabro*; (**B**) *Vespa orientalis*.

Traps were installed by trained personnel on branches of trees or shrubs at a height of 1.5–1.8 m above ground level. The trap (Tap Trap^®^, Carello Roberto, Pianezza, Italy) consisted of a bright yellow plastic cap, visually attractive to hornets, attached to the neck of a standard 1.5 L transparent plastic bottle. The colour of the cap and the bait inside encouraged hornets to enter, while the internal structure prevented their escape and allowed bees and other small non-target insects to exit. In each site, trap triplets were positioned 3 m apart across the site (141 triplets and total 423 bottles). Each bottle of the triplet contained a different bait consisting of two carbohydrate-based baits (canned peaches and beer) and one protein-based bait (commercial cat food). For the carbohydrate-based baits 80–100 g of canned peaches were cut and immersed in 250 mL water and 12–15 g of white sugar were mixed with 250 mL lager beer (5% alcohol); for the protein-based bait 40–50 g of salmon-flavoured wet cat food were homogenised in 250 mL of water. The number of trap triplets deployed at each site varied according to the width of the monitoring site to ensure adequate spatial coverage. Traps were inspected every week for eight following weeks by emptying the bottles on a white absorbable paper and baits were immediately replaced with fresh material of the same type. Captured hornets were morphologically identified and only individuals identified as *V. orientalis* and *V. crabro* were counted and included in the analysis (Figure 2).

### 2.3. Statistical Analysis

Before conducting the inferential analysis, the composition of the sample was assessed using chi-square tests. These tests evaluated the overall species prevalence (*V. crabro* vs. *V. orientalis*), the heterogeneity in species composition between the two sampling environments (apiaries vs. urban sites), and the differences in species prevalence across the bait types used. These preliminary tests were performed to justify the inclusion of Species and Environment as covariates in the main model.

All count data (response variable: Catches) were analysed using a Generalised Linear Model (GLM) specified with a negative binomial distribution (chosen to account for significant overdispersion of the dispersion parameter, Phi) and a logarithmic link function.

Due to non-convergence of the initial complex model, the analysis was conducted using two separate GLMs (Model 1 and Model 2) focused on the fixed effects of interest.

Model 1 tested the primary hypothesis of a differential bait effect depending on the species and used as fixed effects: Bait (Peach = reference), Species (*V. crabro* = reference), and their interaction (Bait × Species).

Model 2 tested the secondary hypothesis of a differential environment effect depending on the species and used as fixed effects: Environment (urban = reference), Species (*V. crabro* = reference), and their interaction (Environment × Species).

Statistical significance for fixed effects was assessed using the Wald chi-square test.

Furthermore, to evaluate the potential influence of the geographic proximity of sampling sites on hornet abundance, a Global Moran’s I test for spatial autocorrelation was performed. This analysis was crucial to ensure the independence of observations, given the geographic clustering of some sites (e.g., the Naples metropolitan area). Spatial relationships were defined using a weight matrix based on the inverse of the Euclidean distance between coordinates (1/dij), ensuring that closer sites had a stronger weight in the autocorrelation calculation.

A significance level of alpha = 0.05 was used for all tests. All statistical analysis was conducted using JMP, version 18.0 (SAS Institute, Cary, NC, USA).

## 3. Results

### 3.1. Hornet Capture Count

Detailed results of the hornet counts, according to bait type and trap location (apiaries and urban sites), are reported in the Tables S1 and S2 of the Supplementary Materials. At the end of the experiment, a total of 419 hornets were captured, of which 268 (64%) were *V. crabro* and 151 (36%) were *V. orientalis*.

The Global Moran’s I test revealed no significant spatial autocorrelation for hornet captures across the study area (I = 0.082; E[I] = −0.062; z = 0.95; *p* = 0.34). These results indicate that the distribution of *Vespa* sp. captures is not significantly clustered or dispersed at a large scale. This suggests that hornet abundance is primarily influenced by site-specific environmental variables or local attractors, such as the presence of apiaries, rather than by a spatial contagion process, confirming the statistical independence of the sampling sites for the GLM analysis. In apiaries 295 hornets were captured. Of these, 69 individuals (23.4%) were identified as *V. orientalis*, while 226 individuals (76.6%) were identified as *V. crabro*. Among the *V. orientalis* specimens, 62 of the 69 individuals (89.9%), were captured in the bottles containing cat food; 4 out of 69 individuals (5.8%) were captured in bottles baited with canned peaches, while 3 of the 69 individuals (4.3%) were captured in the bottles baited with beer. *V. orientalis* was captured only in Naples and Caserta and was absent from apiary samples in Avellino, Benevento, and Salerno. Among the 226 *V. crabro* specimens collected, 33 individuals (14.6%) were captured in traps baited with canned peaches, 179 individuals (79.2%) in those baited with beer, and 14 individuals (6.2%) in traps containing cat food.

In urban sites 124 hornets were captured, of which 82 (66.13%) were identified as *V. orientalis*, while 42 individuals (33.9%) were identified as *V. crabro*.

Regarding the *V. orientalis* specimens, 77 individuals (93.90%) were captured in bottles containing cat food, three (3.66%) in bottles baited with canned peaches, two (2.44%) were captured in bottles containing beer. Regarding *V. crabro* specimens, 26 individuals (61.90) were found in bottles baited with beer, five individuals (11.90%,) in traps baited with canned peaches and 11 individuals (26.19%) in traps containing cat food.

### 3.2. Statistical Results

**Figure 3:**
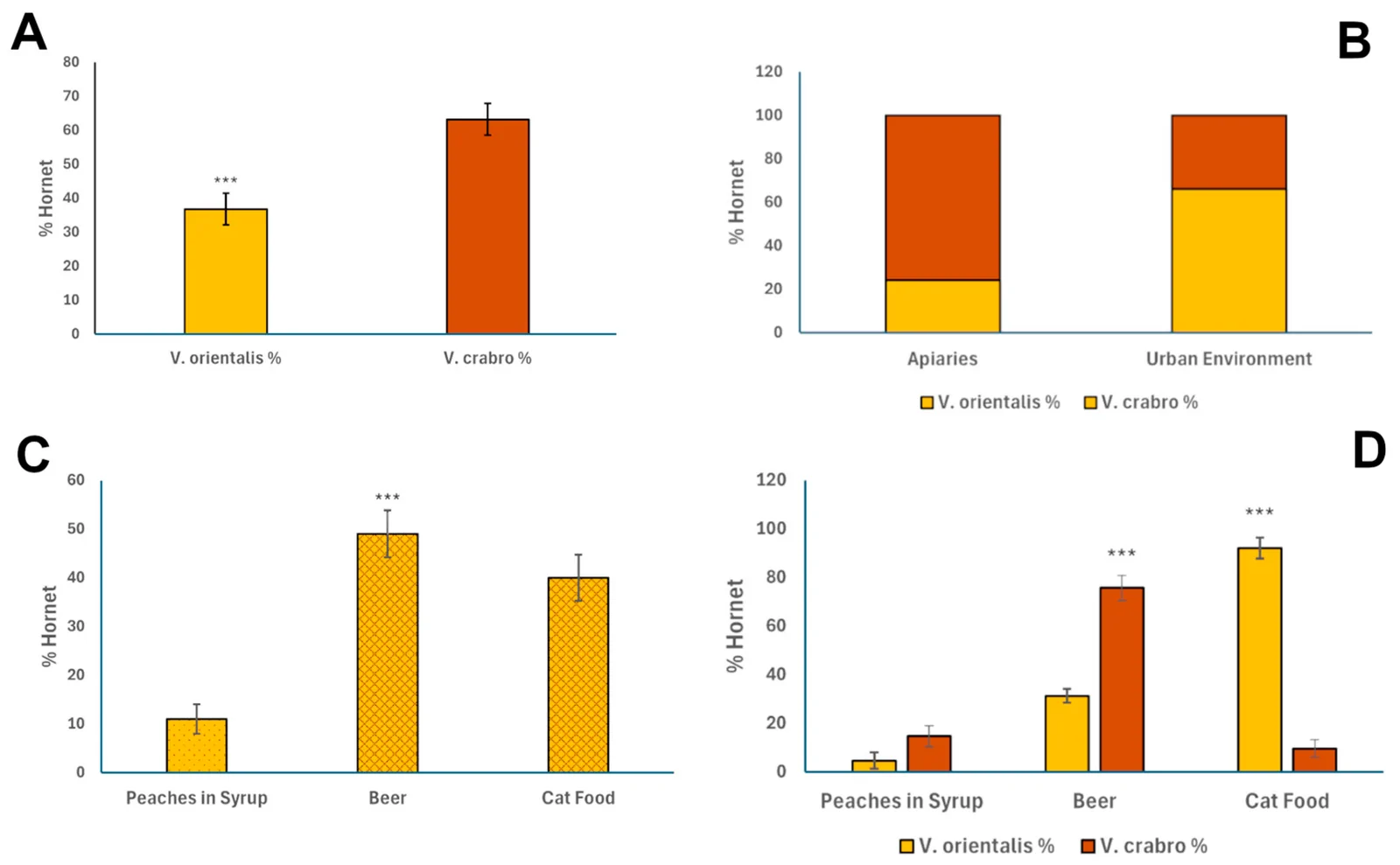
Descriptive analysis of hornet sample composition and bait preference. The figure summarises the aggregate sampling results used to justify the inclusion of covariates in the GLM. (**A**) Species Prevalence: overall proportion of the two sampled species; (**B**) Spatial Distribution: species composition across the two sampled environments (apiaries and urban); (**C**) Aggregate Palatability (Main Effect of Bait): percentage of preference for total baits; (**D**) Species-Specific Palatability: distribution of catches per bait. Data are expressed as percentage ± SE. *** *p* < 0.001.

The overall analysis of the collected hornet population revealed a significant prevalence of *V. crabro* (63.2%) compared to *V. orientalis* (36.8%) (χ^2^ = 63.80, *p* < 0.0001). However, the sample composition was significantly heterogeneous between the two sampled environments (χ^2^ = 145.85, *p* < 0.0001). Specifically, the population was dominated by *V. crabro* (75.8%) in apiaries, while *V. orientalis* (66.1%) was the most captured species in urban environments. This spatial variation in captured species justified the inclusion of the Environment variable in the GLM (Figure 3).

#### Efficacy of Main Factors on Total Catches

The analysis was conducted using two separate GLMs with a negative binomial distribution (N = 150). Table 1 shows that the choice of the negative binomial distribution was justified, as the dispersion parameter (φ) was highly significant in both models (*p* < 0.0001 for both, with Phi for Model 1 = 0.8161). Fixed effects parameter estimates are summarised in Table 2.

Model 1 (testing the Bait × Species interaction) showed that the main effect of Bait was not statistically significant (Wald χ^2^ Prob > χ^2^ = 0.9705). However, the Bait × *V. crabro* interaction was statistically significant (Wald χ^2^ Prob > χ^2^ = 0.0125), indicating that bait effectiveness differs significantly for *V. crabro* (Table 2). The Bait × *V. orientalis* interaction was not significant (*p* = 0.3277).

Post hoc analysis of the significant Bait × *V. crabro* interaction (Wald χ^2^ Prob > χ^2^ = 0.0125) identified the nature of this specificity. The statistical significance is driven by the clear preference shown by *V. crabro* for the beer bait in terms of Log Capture Rate compared to the reference bait (canned peaches). The overwhelming effectiveness of the beer bait for the capture of *V. crabro* demonstrates that bait efficacy is highly species-dependent for *V. crabro*.

Model 2 (testing the Environment × Species interaction) found no significant difference in the total number of catches between the apiary and urban environments (Prob > χ^2^ = 0.2099). In this model, the main effect of Species was also not significant (Prob > χ^2^ = 0.8555). The Environment × Species interaction was also not significant (*p* = 0.9759).

Consequently, data support the hypothesis that bait efficacy acts as a species-specific factor, but this specificity is driven by the significant Bait × *V. crabro* interaction (*p* = 0.0125). The results suggest that bait efficacy is not consistent across all species. The environment did not act as a species-specific factor. Supporting these inferential results, descriptive analysis of bait attractiveness (without considering species) indicated that beer (49%, CI95: 44.19–53.86) and cat food (40%, CI95: 35.26–44.74) were the most frequently chosen baits overall, vastly surpassing canned peach (11%, CI95: 7.95–14.00).

## 4. Discussion

Today *Vespa orientalis* L. is considered the most problematic pest for beekeeping in the Campania Region. As hornets (Hymenoptera: Vespidae) have become unexpectedly abundant in apiaries, numerous trapping and control methods have been tested, and a wide range of baits has been employed [56,58,59,60,61]. Among the various approaches, baited traps have demonstrated notable efficacy. In particular, transparent plastic bottle traps with a coloured cap filled with beer have proven to be the most efficient for capturing several social wasp and hornet species in Europe [51,60,62,63], including *V. crabro* [51]. The same trap, when baited with fish-based proteins, was identified as the most effective for capturing *V. orientalis* [56].

In our study, we employed the Tap Trap^®^ (www.taptrap.com) combined with three types of bait: canned peaches, lager beer mixed with sugar, and salmon-flavoured wet cat food diluted with water. Hornet attraction was assessed by counting the number of individuals captured in each trap, with the most effective bait defined as the one yielding the highest number of captures. Our results, consistent with Demichelis [51], confirm that beer is the most attractive bait for *V. crabro*, followed by peaches and fish-based cat food, with significant differences among them. Conversely, *V. orientalis* did not show a clear preference for any of the tested baits, as indicated by the absence of significant differences among the three baits. However, it still appears that *V. crabro* workers were more interested in sugar baits than in protein baits, whereas *V. orientalis* workers seemed to be still interested in the protein baits more than in the sugar baits. Given the different uses of food sources for Vespids (proteins for larvae and carbohydrates for workers), it is possible that the colonies of the two species were in different moments of their life cycles, with *V. crabro* colonies developing earlier than *V. orientalis* during the fall season. This is in line with our observations of the past years in which we noted a delay in the appearance of males and foundresses of *V. orientalis*. Thus, we can suggest that during fall, adults of *V. crabro* need to forage sugary foods for their survival, while *V. orientalis* larvae need to collect higher amounts of proteins for the correct development of larvae. On the other hand, compared to *V.orientalis*, observations on *V. crabro* foraging behaviour have shown that workers return to their nests carrying prey in approximately 1% of flights, indicating a limited reliance on proteinaceous resources. Therefore, it could be possible that this species is more likely to rely on carbohydrate rich substrates over protein-based baits [10]. Additionally, sweetened beer emits volatile organic compounds analogous to those produced by ripe or fermenting fruits, which are known to function as potent attractants for *V. crabro*.

Nevertheless, more details are needed to confirm these hypotheses.

Although bait preferences may vary geographically [64], our findings—in agreement with Demichelis et al. [51]—show that the same traps and baits deployed in different environments (apiary and urban sites) did not differ in their attractiveness to the captured hornet species. However, the number of hornets captured varied across different environments as *V. crabro* predominated in apiaries, whereas *V. orientalis* was more common in urban areas, highlighting the strong tendency of *V. orientalis* to colonise synanthropic habitats. This pattern may reflect differences in the surrounding landscape, as urban environments provide abundant anthropogenic food resources and nesting opportunities that may favour *V. orientalis*, whereas more rural contexts may support *V. crabro* and its preference towards wild insects.

Despite the high presence of *V. orientalis* in apiaries, its low capture rates suggest that current trapping systems and attractants are poorly suited to this species [65]. This discrepancy likely reflects behavioural traits: *V. orientalis* appears to visit apiaries primarily to prey on honey bees rather than to exploit general food resources [47]. Trap baits—often difficult to access and characterised by weak or unattractive odours—are unlikely to compete with the strong olfactory and visual cues emitted by honey bee colonies. Experimental work shows that *V. orientalis* exhibits selective feeding responses with many bait types being insufficiently attractive and rapidly losing efficacy [66,67]. Bees are concentrated at hive entrances, offer minimal defensive response, and their movement strongly stimulates predatory behaviour, making them far more attractive than static baits [47].

Trap placement may further limit capture efficiency. Studies comparing trap heights and positions demonstrate that traps placed away from hive entrances or at suboptimal heights capture significantly fewer individuals [68]. In this study traps were placed at 1.50–1.80 m of height to help prevent external manipulation that could interfere with the experiment. However, it is possible that the positioning was too high when compared to previous data which described highest number of catches at 1 m [68]. Therefore, we believe that this element should be further investigated to validate this point.

These findings indicate that conventional attractant-based trapping protocols are inadequate for monitoring or controlling *V. orientalis* in apiary [65]. Future research should prioritise the development of attractants that better mimic the trophic cues associated with predation, such as protein-based signals resembling brood odours or hive distress volatiles. Additionally, trap designs and placements should be reconsidered, with emphasis on positioning devices directly at hive entrances or along flight paths associated with predatory activity.

Overall, our results highlight the need for a shift from generalised attractant-based trapping toward behaviourally informed monitoring tools. A deeper understanding of the ecological and behavioural traits of *V. orientalis* will be essential for designing integrated management strategies capable of accurately detecting, quantifying, and mitigating its impact on honey bee colonies and apicultural productivity.

## Figures and Tables

**Table 1:** Dispersion parameter (Phi) estimates obtained from the two GLMs with a negative binomial distribution. The dispersion parameter is a measure of unexplained overdispersion, and its significance justifies the use of the negative binomial over a Poisson model. ** *p* < 0.01.

Variance Component	Reference Model	Estimate (Phi)	Std Error	Wald *p*-Value
Phi (Model 1)	Bait × Species	0.8161	0.1945	<0.0001 **
Phi (Model 2)	Environment × Species	0.8173	0.1945	<0.0001 **

**Table 2:** Fixed effects test results obtained from two separate Generalised Linear Models (GLM) with a negative binomial distribution (Model 1: Bait × Species; Model 2: Environment × Species). The Wald chi-square test was used to evaluate the overall significance of the factor or interaction on the Log (Capture Rate).

	Model	Nparm	DF	Wald Chi Square	Prob > Chi Square
Interaction bait × *V. crabro*	Model 1	2	2	8.759	0.0125
Interaction bait × *V. orientalis*	Model 1	2	2	2.231	0.3277
Main Effect Bait	Model 1	2	2	0.060	0.9705
Main Effect *V. crabro*	Model 1	1	1	0.037	0.8481
Main Effect *V. orientalis*	Model 1	1	1	0.033	0.8555
Interaction Environment Species	Model 2	1	1	0.001	0.9759
Main Effect Environment	Model 2	1	1	1.577	0.2099

## Data Availability

Research data can be found in archived datasets generated during the study.

## Supplementary Materials

The following supporting information can be downloaded at: https://www.mdpi.com/article/10.3390/insects17040368/s1, Table S1: Monitoring of *Vespa orientalis* (VO) and *Vespa crabro* (VC) in apiaries of Campania region; Table S2: Monitoring of *Vespa orientalis* (VO) and *Vespa crabro* (VC) in urban sites of Campania region.

## Abbreviations

The following abbreviations are used in this manuscript:

VO	*Vespa orientalis*
VC	*Vespa crabro*
EVOC	PROGETTO EMERGENZA VESPA ORIENTALIS IN CAMPANIA II
