# Supplementary material for: Evaluation of Bait Attractiveness for Vespa orientalis and Vespa crabro (Hymenoptera: Vespidae) in Urban and Apiary Environment of Campania Region (Italy)
Source: Insects. 2026 Mar 31;17(4):368. doi: 10.3390/insects17040368 (PMC13115923; doi:10.3390/insects17040368)
Supplement: Supplementary file 1 [file insects-17-00368-s001.zip › Table S1.pdf]

Table S1. Monitoring of *Vespa orientalis* (VO) and *Vespa crabro* (VC) in apiaries of Campania region.

Province	Geographic Coordinates (Lat, Lon) and Landscape context	Bait with peaches		Bait with beer		Bait with cat food	
		VO	VC	VO	VC	VO	VC
**AVELLINO**	41.2350, 15.0077 Rural	0/6Traps	11/6Traps	0/6Traps	41/6Traps	0/6Traps	0/6Traps
	40.9360, 15.1710 Rural	0/3Traps	0/3Traps	0/3Traps	4/3Traps	0/3Traps	0/3Traps
**BENEVENTO**	41.32565, 14.76066 Rural	0/4Traps	1/4Traps	0/6Traps	7/6Traps	0/6Traps	0/6Traps
	41.1280, 14.7800 Rural	0/4Traps	5/4Traps	0/6Traps	13/6Traps	0/6Traps	2/6Traps
**CASERTA**	41.1995, 14.1964 Rural	0/1Traps	0/1Traps	0/2Traps	0/2Traps	0/2Traps	0/2Traps
	41.219559,14.161050 Rural	0/2Traps	0/2Traps	1/4Traps	0/4Traps	0/4Traps	0/4Traps
**SALERNO**	40.7833, 14.7572 Rural	0/4Traps	0/4Traps	0/4Traps	0/4Traps	0/4Traps	0/4Traps
	40.7516, 14.6550 Rural	0/4Traps	3/4Traps	0/4Traps	9/4Traps	0/4Traps	0/4Traps
**NAPLES**	40.8887, 14.2425 Urban	3/9Traps	1/9Traps	0/9Traps	11/9Traps	7/9Traps	6/9Traps
	40.8306, 14.1003 Urban	1/6Traps	1/6Traps	2/6Traps	11/6Traps	17/6Traps	0/6Traps
	40.8727, 14.3021 Urban	0/4Traps	1/4Traps	0/4Traps	2/4Traps	0/4Traps	3/4Traps
	40.8732, 14.2650 Urban	0/4Traps	1/4Traps	0/4Traps	12/4Traps	0/4Traps	2/4Traps
	40.9791, 14.3828 Urban	0/4Traps	0/4Traps	0/4Traps	0/4Traps	0/4Traps	0/4Traps
	40.8407, 14.0968 Urban	0/9Traps	5/9Traps	0/9Traps	10/9Traps	0/9Traps	0/9Traps
	40.8006, 14.3567 Urban	0/9Traps	4/9Traps	0/12Traps	59/12Traps	38/12Traps	1/12Traps
