# Supplementary material for: Evaluation of Bait Attractiveness for Vespa orientalis and Vespa crabro (Hymenoptera: Vespidae) in Urban and Apiary Environment of Campania Region (Italy)
Source: Insects. 2026 Mar 31;17(4):368. doi: 10.3390/insects17040368 (PMC13115923; doi:10.3390/insects17040368)
Supplement: Supplementary file 1 [file insects-17-00368-s001.zip › Table S2.pdf]

Table S2. Monitoring of *Vespa orientalis* (VO) and *Vespa crabro* (VC) in Urban sites of Campania region

Province	Geographic Coordinates (Lat, Lon) and Landscape context	Bait with peaches		Bait with beer		Bait with cat food	
		VO	VC	VO	VC	VO	VC
**NAPLES**	40.8607,14.2633 Urban	0/3Traps	0/3Traps	0/9Traps	2/9Traps	15/9Traps	0/9Traps
**NAPLES**	40.8250,14.2430 Urban	1/9Traps	2/9Traps	1/10Traps	7/10Traps	33/10Traps	5/10Traps
**NAPLES**	40.8490,14.2430 Urban	0/6Traps	0/6Traps	1/12Traps	1/12Traps	6/12Traps	1/12Traps
**NAPLES**	40.8126,14.3407 Urban	2/9Traps	3/9Traps	0/9Traps	5/9Traps	18/9Traps	5/9Traps
**NAPLES**	40.8200,14.3380 Urban	0/2Traps	0/2Traps	0/4Traps	3/4Traps	0/4Traps	0/4Traps
**NAPLES**	40.8230,14.3390 Urban	0/2Traps	0/2Traps	0/4Traps	2/4Traps	0/4Traps	0/4Traps
**NAPLES**	40.8710,14.2420 Urban	0/3Traps	0/3Traps	0/3Traps	0/3Traps	0/3Traps	0/3Traps
**NAPLES**	40.7430,14.4890 Suburban	0/4Traps	0/4Traps	0/6Traps	2/6Traps	0/6Traps	0/6Traps
**NAPLES**	40.7710,14.4730 Suburban	0/4Traps	0/4Traps	0/6Traps	1/6Traps	0/6Traps	0/6Traps
**NAPLES**	40.8173,14.3523 Suburban	0/4 Traps	0/4 Traps	0/6Traps	3/6Traps	5/6Traps	0/6Traps
